# SPdb – a signal peptide database

**DOI:** 10.1186/1471-2105-6-249

**Published:** 2005-10-13

**Authors:** Khar Heng Choo, Tin Wee Tan, Shoba Ranganathan

**Affiliations:** 1 Department of Biochemistry, National University of Singapore, Singapore; 2 Department of Chemistry and Biomolecular Sciences & Biotechnology Research Institute, Macquarie University, Sydney, Australia

## Abstract

**Background:**

The signal peptide plays an important role in protein targeting and protein translocation in both prokaryotic and eukaryotic cells. This transient, short peptide sequence functions like a postal address on an envelope by targeting proteins for secretion or for transfer to specific organelles for further processing. Understanding how signal peptides function is crucial in predicting where proteins are translocated. To support this understanding, we present SPdb signal peptide database , a repository of experimentally determined and computationally predicted signal peptides.

**Results:**

SPdb integrates information from two sources (a) Swiss-Prot protein sequence database which is now part of UniProt and (b) EMBL nucleotide sequence database. The database update is semi-automated with human checking and verification of the data to ensure the correctness of the data stored. The latest release SPdb release 3.2 contains 18,146 entries of which 2,584 entries are experimentally verified signal sequences; the remaining 15,562 entries are either signal sequences that fail to meet our filtering criteria or entries that contain unverified signal sequences.

**Conclusion:**

SPdb is a manually curated database constructed to support the understanding and analysis of signal peptides. SPdb tracks the major updates of the two underlying primary databases thereby ensuring that its information remains up-to-date.

## Background

Günter Blobel discovered that "proteins have intrinsic signals that govern their transport and localization in the cell" [1]. Proteins synthesised at the ribosome (cytoplasm or rough endoplasmic reticulum), mitochondria or chloroplast are transported to their site of function. This process is known as protein targeting and it depends on targeting signals to direct the proteins to their specific locations.

There are many different classes of targeting signals. One of the commonly occurring signals is formed by short, transient peptides known as signal peptides or leader sequences, which are usually found at the amino terminus of secreted proteins. Signal peptides are present in both prokaryotic and eukaryotic cells, indicating its ancient universal origins. They function like a postal address label on an envelope by targeting the proteins for secretion or to specific organelle for further processing. The signal peptides are cleaved off and degraded upon reaching their targeted locations. Interestingly, not all proteins possess signal peptides [2,3], suggesting that other mechanisms for protein targeting exist.

Over the years, several prediction tools [4-10] have been developed to predict the cleavage sites of signal peptides. These prediction tools require training and testing datasets. As a preparatory step for prediction work, researchers often devote considerable time sifting through primary databases such as Swiss-Prot [11], EMBL [12] and other databases to collate and construct their own datasets. This repetitive process can and should be eliminated by creating a centralised repository of signal peptide sequences.

Searching through popular search engines and reviewing the Nucleic Acids Research database list [13] reveal several databases that provide information on protein subcellular localisation [14,15], nuclear proteins [16] and secreted proteins [17]. These databases do not provide signal peptide specific information except for SPD [17]. SPD or secreted protein database [17] is a collection of proteins from the human, mouse and rat proteomes originating from databases such as TrEMBL [18], Ensembl [19] and Refseq [20]. It also includes datasets from the Secreted Protein Discovery Initiative (SPDI) [21], a large-scale effort to identify novel human secreted and transmembrane proteins; the Riken mouse secretome and seven other related datasets [22]. SPD aims to be a comprehensive repository for secreted proteins, but it suffers from providing datasets that may still contain many erroneous annotations from its underlying data sources for example TrEMBL. TrEMBL is generated from an automated pipeline and has yet to undergo manual curation. In addition, the entries in SPD were not checked manually against the publications. Then, there is also the issue that the datasets are not being updated.

Besides the SPD which offer downloadable datasets, there are sites that offer downloadable datasets namely the SignalP datasets (1997) [10,23] and the datasets (2000) used by Meene *et al*. [24,25] in their evaluation of signal peptide prediction methods. More recently, there is the dataset consisting of 270 secreted recombinant human proteins with experimentally determined cleavage sites from Zhang and Henzel [26,27]. These datasets are often either limited in size or otherwise lacking in tools for querying the datasets. Moreover, these datasets although valuable but they are often outdated [28] especially when GenBank/EMBL, Swiss-Prot and other publicly accessible primary databases continue to churn out new entries or sequences.

Many researchers are confronted by similar obstacles in accessing up-to-date data, which are withheld from public access by method developers [29,30] and hence, we strongly believe that there is a urgent need to provide a publicly-accessible, manually curated and regularly updated database specialised for signal peptides. These datasets will not only be important for prediction work but they will also serve as the common datasets needed when researchers are performing benchmarking against each other methods or programs, without which we think it is difficult to perform proper or fair benchmarking of the multitudes of prediction methods.

## Construction and content

### Construction and implementation of database

Recognizing the need for a curated, specialised and up-to-date database, we have developed a composite signal peptide database SPdb [31] that offers researchers a singular point for depositing their signal peptide annotations. SPdb integrates information from Swiss-Prot (part of UniProt) [18] and EMBL. It is updated when there is a major release of Swiss-Prot. First released in May 2004, SPdb was upgraded to release 3.2 recently to add on new features and to synchronise with the release of its underlying data sources.

SPdb is a relational database built using MySQL database management system [32] and using PERL/CGI [33] for processing web forms. An easy-to-navigate web interface was built to allow user to search through the database. Some of the web features were added in response to the requests by some of the users that have used our database since its inception. Through the web interface, users are able to download the returned results as FASTA formatted files or view the results as HTML web page. We have also provided a link from the search page to the Swiss-Prot ID tracker to verify whether an entry has been renamed e.g. ANL3_HUMAN from the Zhang and Henzel dataset [27], which is now ANGL3_HUMAN.

**Figure 1:**
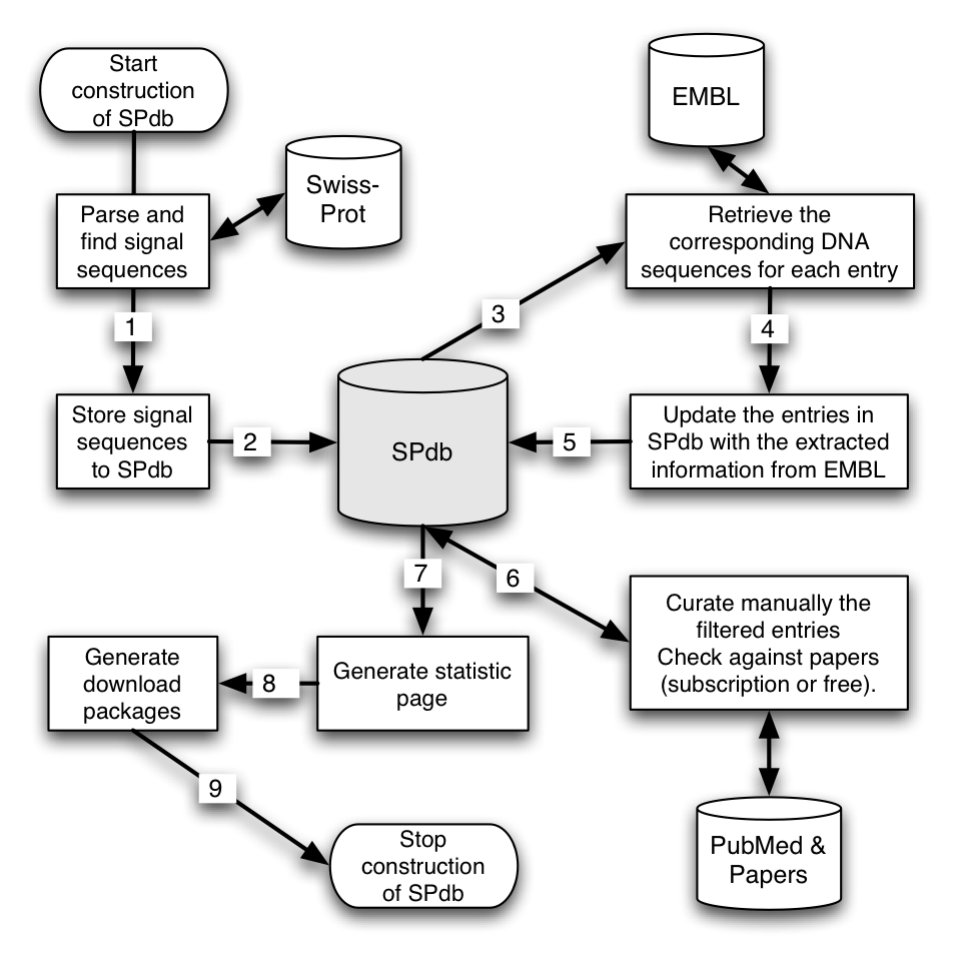
Schematic diagram of the construction pipeline of SPdb.

We deployed the bioinformatics pipeline shown in (Figure 1) to construct SPdb. The pipeline to construct the database was semi-automated with specific checkpoints for manual checking of the results to minimise errors in the database.

### Construction method

Signal sequences and coding sequences obtained from Swiss-Prot (TrEMBL entries were not taken into consideration) were filtered initially using the data extraction and redundancy reduction methodology proposed by Nielsen *et al*. [34] to segregate the dataset into two sets (a) the *preliminary filtered *set and (b) the *unverified sequences *set. The Nielsen *et al*. [34] methodology has been employed to generate the training and testing data used in SignalP [10,35]. We adapted and omitted some of the criteria proposed by the method since our goal is to build a repository of signal peptides with as many relevant and accurate entries as possible. We observed that the proposed methodology still renders many undesirable entries upon the filtering process. Thus, we have constructed SPdb by building on the strength of the proposed methodology [34] and improved it with our own criteria and filtering rules.

Any entries with the SIGNAL keyword indicated in the feature table FT field [36] of Swiss-Prot entries were presumed to contain information on signal sequence. This simple selection process yielded 18,146 entries out of the total 170,140 Swiss-Prot entries (Release 46.1). Entries that connoted uncertainty namely those with annotations like PROBABLE, POTENTIAL, BY SIMILARITY, HYPOTHETICAL and entries with ambiguous cleavage or signal peptide positions were tagged as *unverified sequences*. Then, entries with signal sequences length less than 11 were relegated to the *unverified sequences *set. Signal sequences are generally considered to be of length 15 to 40. This initial step filtered off 13,701 entries from the *preliminary filtered *set leaving behind 4,445 entries. These entries include type I signal peptides, type II signal peptides (lipoproteins) and TAT-containing signal peptides. Using the SIGNAL keyword, mitochondria and chloroplast transit peptides were excluded from the *preliminary set *since transit peptides are identified by the TRANSIT keyword in Swiss-Prot.

We proceeded to integrate information from the EMBL database. By integrating complementary information, besides providing extra information not found in Swiss-Prot, we could use the information from EMBL to cross check against Swiss-Prot, allowing us to discover erroneous annotations. This practice of using complementary information from other data sources has been found useful in data evaluation [37].

The first cross-reference entry to EMBL database was used for the respective Swiss-Prot entry. Based on the data categorisation of EMBL found in its release note [38], only sequences from the data groups fungi, human, invertebrate, mouse, organelle, bacteriophage, plant, prokaryote, rodent, viral, mammals and vertebrate were taken into consideration. Entries belonging to the data groups expressed sequence tags, genome survey sequences, high-throughput genome sequences, unfinished DNA sequences generated by high-throughput sequencing, patent sequences, synthetic sequences, contig sequences and unclassified were omitted. We extracted out relevant annotations from EMBL whenever available including coding sequence, signal sequence and its length, subcellular location, authors' notes and so on.

The annotations, specifically the *sig_region *and *misc *fields from the EMBL entry were utilised in the subsequent step to cross-check against the *preliminary filtered *entries. This step again filtered out many inconsistent entries where the positions are quoted wrongly by either source e.g. [Swiss-Prot:CD166_CHICK] where Swiss-Prot quoted cleavage position of 33 while EMBL provided 32. As a result, another 866 entries were eliminated to retain 3,579 entries in this newly filtered *Swiss-Prot/EMBL *set. It must be noted that there were some Swiss-Prot entries in the *Swiss-Prot/EMBL *set without any EMBL reference e.g. [Swiss-Prot:APOE_CAVPO]; or with insufficient annotations in the EMBL entries e.g. [Swiss-Prot:17KD_RICAU]; or their EMBL cross-references were indicated with annotation such as NOT_ANNOTATED_CDS e.g. [Swiss-Prot:2B31_HUMAN], ALT_TERM e.g. [Swiss-Prot:CD1E_HUMAN], ALT_INIT e.g. [Swiss-Prot:1A03_PANTR] and ALT_SEQ e.g. [Swiss-Prot:17KD_RICPR]. In these cases, all these entries were earmarked for manual curation. These terms "NOT_ANNOTATED_CDS", "ALT_TERM" and so on are known as *status identifiers *and they are found at the DR field in Swiss-Prot entries. The reader is referred to the detailed explanation found in the Swiss-Prot manual [39].

Following this step, all the entries in this *Swiss-Prot/EMBL *set are manually checked against the referred publications. We located numerous entries with discrepancies on signal peptide cleavage site between the Swiss-Prot annotations and the accompanying papers e.g. [Swiss-Prot:CECC_DROME, Swiss-Prot:AMCY_PARVE]. Entries that we do not have access to the accompanying papers e.g. [Swiss-Prot:ZEAL_MAIZE, Swiss-Prot:ZEA6_MAIZE] or those entries that we could not locate their cleavage site information in the papers e.g. [Swiss-Prot:GUX1_TRIRE]; these entries in addition to those entries which are inadequately labelled or entries with inconsistent positional information were all relegated to the *unverified sequences *set. In this manual curation step, we eliminated 995 entries from the *Swiss-Prot/EMBL *set of 3,579 entries. These 995 entries were entries that (a) both Swiss-Prot and the quoted papers provided the same putative position (b) we found differing positions quoted by Swiss-Prot as compared to the quoted papers (c) we did not have access to the quoted subscription-only papers or the papers referred to were old and in some cases there were no paper or no relevant paper quoted (d) we could not find or locate the cleavage site information (Table 1).

**Table 1:** Distribution of the signal sequences filtered out in the manual curation step

Description	No. of Entries/Sequences
Swiss-prot and the accompanying papers quoted same putative position	311
Swiss-Prot and the accompanying papers quoted different position; The position quoted maybe confirmed or putative	100
No references or relevant references were provided; No access to some subscription-only papers; No access to some very old papers	194
Unable to locate or obtain the position information from the papers	390
TOTAL	995

### Content of database

The result from filtering and manually curating the entries culminated in the latest release of SPdb release 3.2, with a total of 18,146 signal sequence entries, out of which 2,584 are filtered sequences (Table 2). These filtered sequences, known as the *filtered sequences *set include the mature endogenous proteins that were sequenced on their N-terminal and have been checked against the accompanying reference paper to be considered as experimentally verified positions. The remaining 15,562 *unverified sequences *contain putative or experimentally unverified cleavage site signal sequences. This *unverified *set also contains entries with erroneous database annotations. It is worth noting that this *unverified *set might contain some experimentally verified signal sequences since we may not have access to the accompanying papers.

**Table 2:** Distribution of signal sequences in SPdb according to archaea (AR), bacteria (BA), viruses (VR) and eukaryotes (EU).

	AR	BA	EU	VR	SUB-TOTAL
Verified sequences	7	540	1,945	92	2,584
Unverified sequences	101	3,528	11,239	694	15,562
TOTAL	108	4,068	13,184	786	18,146

**Figure 2:**
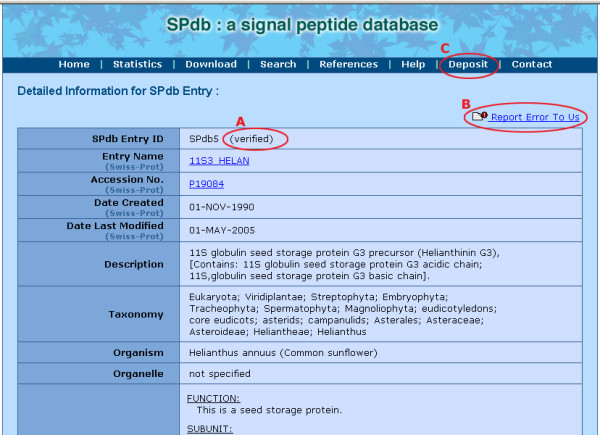
SPdb entry information includes a short description of the protein, the hydropathy plots and amino acids properties and more. (A) Each entry is marked as verified or unverified, with (B) a "report-error" link for users to inform us on any error or updated information pertaining to an entry for us to rectify/update. (C) users can deposit their signal sequences with us and add on their own annotation.

With the two primary databases integrated, SPdb contains four data groups namely archaea, bacteria, eukaryotes and viruses and (Table 2). SPdb provides key extracted information (Figure 2) such as organism source, organelle, subcellular location and other accompanying important notes. For full annotation, cross-referenced links to the originating database are provided. The signal peptide cleavage site is explicitly marked if such information is available. The signal peptide sequences and 30 residues [40] after the cleavage site are colour-coded using the convention as specified by RasMol amino acids colour scheme [41] which is based on the traditional amino acid properties. In the process of manually curating the 3,579 entries, we have added our own annotation for the 995 entries that were removed from this dataset later on.

## Utility and discussion

SPdb provides users with an easy-to-use web interface with flexibility to select for an entry or a collective set of entries matching users' criteria such as name of organism, data group, length of signal sequences, keyword searches and more importantly the option to choose between including or excluding certain entries. We have taken the approach to allow users to omit or filter any sequences since every user may have different requirements on the returned results. Each of the entry will be indicated whether it is verified or unverified (Figure 2).

In the process of creating SPdb, we realise that although Swiss-Prot provides better quality annotation, it still contains erroneous or conflicting annotations as evident when we compare the positions or length of the signal sequences reported by Swiss-Prot with EMBL e.g. [Swiss-Prot:A2AP_HUMAN, Swiss-Prot:BTD_HUMAN]. We notice that the inconsistencies usually arise when there is more than one reference. The referred papers may quote different positions thus this may have caused the confusion. To help to resolve this issue, we have combined the annotations from -EMBL, and we managed to identify and filter off many such entries. The annotations on signal peptide found in EMBL were mostly accurate though there were cases when the information was reported incorrectly as well e.g. [EMBL:M19077] in [Swiss-Prot:CHR1_BOMMO]. In this respect, we have included a link in each entry for users to report to us if they encounter any errors or discrepancies in SPdb.

Apart from the errors and inconsistencies just described, there was also the issue on experimental support from the journal publication. Many of the entries with annotation on signal sequences positions or length were predicted or deemed putative or potential (Table 1) by the researchers when they reported the positions in their papers. Nonetheless, the entries were not labelled with words like POTENTIAL, BY SIMILARITY and PROBABLE in the Swiss-Prot entry as previously assumed. We learnt that many of the referred papers were using prediction or sequence alignment software to identify or suggest the cleavage site of signal sequences. Therefore, we think it would be more appropriate and useful if the references to the papers were also indicated at the relevant fields so that any users of the entries can easily check and read up on the papers that mentioned about signal peptides or any other features.

All these issues and problems have made automation of the construction of SPdb immensely difficult if not remotely impossible. Prior to manually curating the entries, we have considered using text-mining approach but we forwent the method eventually when we discovered that many of abstracts did not contain the cleavage site information rather the information was found in the body of the paper, usually located under the results or discussion section. Moreover, the words or phrases used to express the positional information were also varying and difficult to express as extraction rules e.g. in the paper [42] quoted in entry [Swiss-Prot:PRRP_BOVIN], we encountered this sentence "... its N-terminal portion before Ser-23 showed the typical profile of a secretory signal peptide ...". Then there is also the problem where many of the papers require subscriptions, rendering the extraction program useless unless we can obtain the papers. Unless each of the paper submitted in future provides a short note on the features of the proteins described coupled with the improvement in text-mining accuracy, we will have to resort to manual curation.

In SPdb, datasets are classified into *filtered sequences *and *unverified sequences*. By classifying the entries into these two classifications, researchers can use them in the work of machine learning approaches, where datasets are sought after as training and testing sets in signal peptide cleavage site prediction.

Apart from facilitating test datasets, SPdb provides other information such as amino acid composition of the protein which have been suggested to correlate with the subcellular localisation of the protein [43]; amino acid residues properties (aromatic, non-polar, polar, charged and so on) are shown in graphical format to indicate which residues possess the properties visibly; also accompanying each entry are the hydropathy plots based on Kyte and Doolittle [44], Sweet and Eisenberg [45], Eisenberg *et al*. [46] of the signal sequences and the sequences downstream of the signal sequence cleavage. The plots are rendered using pepinfo within the computational analysis package of EMBOSS [47], an open source software suite for sequence analysis. Each signal peptide exhibits three distinct regions at the sequence level: the *n-region *(a positive charged region), the *h-region *(hydrophobic region) and the *c-region *(polar and neutral region) [9]. The hydropathy plots help to visualize and identify these regions.

De Gier *et al*. [48] showed that signal peptide processing by the signal recognition particle (SRP) requires certain contextual cues in the sequence downstream. SRP binds to N-terminus signal or signal-anchor sequence when the nascent polypeptide chain is synthesised by the ribosome up to ~60 amino acid residues. At this length, this segment is conveniently exposed and translation will resume upon dissociation of SRP from the nascent chain. In the effort to capture this information for the co-translational translocation mechanism, SPdb includes both the signal peptide sequences and 30 residues after the cleavage site.

For the future releases, we hope to include other information which maybe useful such as functional classification of signal peptides according to target destination, the profiles of signal peptides from various organisms and so on. As differentially targeted organelles or locations have variations on the general theme of signal peptide target proteins, we would like to include these different targeting signals for comparison and studies. Concerning secreted proteins that lack cleavable signal peptide [49] e.g. ovalbumin, a secreted glycoprotein and the major protein of egg-white which does not have a cleavable signal peptide [50], we would like to include this information and analyse how they differ from those proteins with cleavable signal peptide.

## Conclusion

Signal peptide plays an important role in the transport of secretory proteins. Understanding of signal peptide recognition and mechanisms of targeting, transport and translocation will unleash many applications in the area of drug design and medicine. We have provided a freely accessible, manually curated signal peptide database that is regularly updated and synchronised with the release of the two major primary databases, Swiss-Prot and EMBL. By integrating information from both databases, SPdb is able to eliminate some of the discrepancies and minimise the errors found in the sequence entries, thereby providing a better quality of the downloadable datasets that can be used by the research community for prediction work and other research.

## Availability and requirements

SPdb is freely accessible through the website . We have made available a dedicated page to allow user to download the dataset in full based on certain criteria available to user.

## Authors' contributions

KHC built the database pipeline and the web interface. SR developed the signal peptide project and provided comments and suggestions on the features of the database while TWT provided assistance for the database design and the manuscript.
